# ID30B – a versatile beamline for macromolecular crystallography experiments at the ESRF

**DOI:** 10.1107/S1600577518007166

**Published:** 2018-06-27

**Authors:** Andrew A. McCarthy, Ray Barrett, Antonia Beteva, Hugo Caserotto, Fabien Dobias, Franck Felisaz, Thierry Giraud, Matias Guijarro, Robert Janocha, Akim Khadrouche, Mario Lentini, Gordon A. Leonard, Marcos Lopez Marrero, Stephanie Malbet-Monaco, Sean McSweeney, Didier Nurizzo, Gergely Papp, Christopher Rossi, Jeremy Sinoir, Clement Sorez, John Surr, Olof Svensson, Ulrich Zander, Florent Cipriani, Pascal Theveneau, Christoph Mueller-Dieckmann

**Affiliations:** a European Molecular Biology Laboratory, Grenoble Outstation, 71 avenue des Martyrs, Grenoble 38042, France; b European Synchrotron Radiation Facility, 71 avenue des Martyrs, Grenoble 38043, France

**Keywords:** ID30B, MD2S, REX rapid nozzle changer, FlexHCD, SPINEplus gripper, experimental phasing, automation

## Abstract

ID30B is an undulator-based high-intensity, energy-tuneable (6.0–20 keV) and variable-focus (20–200 µm in diameter) macromolecular crystallography (MX) beamline at the ESRF. It was the last of the ESRF Structural Biology Group’s beamlines to be constructed and commissioned as part of the ESRF’s Phase I Upgrade Program and has been in user operation since June 2015. Both a modified microdiffractometer (MD2S) incorporating an *in situ* plate screening capability and a new flexible sample changer (the FlexHCD) were specifically developed for ID30B. Here, the authors provide the current beamline characteristics and detail how different types of MX experiments can be performed on ID30B (http://www.esrf.eu/id30b).

## Abstract 2

ID30B, a versatile macromolecular crystallography beamline at the ESRF, is presented.

## 1. Introduction   

ID30B was the last of the facilities to be constructed and commissioned as part of the UPBL10 project of the ESRF Phase I Upgrade Program and is the most recent addition to the Joint Structural Biology Group (JSBG) portfolio of beamlines at the ESRF (Mueller-Dieckmann *et al.*, 2015 ▸). The UPBL10 project was conceived to replace and supersede the highly automated and successful ESRF ID14 beamline complex (Wakatsuki *et al.*, 1998 ▸), and comprises two fixed-energy macromolecular crystallography (MX) beamlines [ID30A-1 (Bowler *et al.*, 2015 ▸) and ID30A-3], one tuneable MX beamline (ID30B), and a biological small-angle scattering (bioSAXS) beamline (BM29; Pernot *et al.*, 2013 ▸) all located around the ESRF straight section ID30 (Theveneau *et al.*, 2013 ▸). One of the initial concepts of UPBL10 was to develop one branch (ID30A) of a canted undulator setup as a Massively Automated Sample Selection Integrated Facility (MASSIF) for automated sample evaluation to provide an efficient pipeline of well characterized samples for more tailored data collection on other more dedicated MX beamlines, including ID30B to be situated on the second branch. However, advances in both detector (Broennimann *et al.*, 2006 ▸) and robotic technologies (Nurizzo *et al.*, 2016 ▸; Papp, Felisaz* et al.*, 2017 ▸) significantly advanced during this period and meant that moving samples from a screening beamline to a data collection beamline was no longer a major advantage in terms of either time or data quality. The final incarnation of ID30, based on our previous experience in providing high-quality X-ray beams (Flot *et al.*, 2006 ▸), in instrumentation development (Perrakis *et al.*, 1999 ▸; Cipriani *et al.*, 2006 ▸) and in the development of software needed to collect high-quality diffraction data (Gabadinho *et al.*, 2010 ▸; Bourenkov & Popov, 2010 ▸) is a suite of complementary high-performance MX beamlines providing fully automated data collection and screening on ID30A-1 (Svensson *et al.*, 2015 ▸; Bowler *et al.*, 2016 ▸) and more tailored data collection possibilities on ID30A-3 and ID30B. These new beamlines expand the MX portfolio at the ESRF (Mueller-Dieckmann *et al.*, 2015 ▸), which are complemented by a modern bioSAXS beamline (Pernot *et al.*, 2013 ▸) located on BM29 and a Titan Krios cryo-electron microscope (CM01).

UPBL10 first improved the ID14 principle (Wakatsuki *et al.*, 1998 ▸) by decoupling the highly automated MASSIF-1 (Bowler *et al.*, 2015 ▸) and mini-beam (MASSIF-3) fixed-energy beamlines on the ID30A branch from the energy-tuneable ID30B branch. This was achieved by using a canted undulator setup similar to that pioneered on ESRF MX beamline ID23 (Nurizzo *et al.*, 2006 ▸; Flot *et al.*, 2010 ▸) that allows both branches to work independently. The second improvement was to build on the high-level automation developed by the JSBG over more than a decade (Arzt *et al.*, 2005 ▸; Beteva *et al.*, 2006 ▸; Brockhauser *et al.*, 2012 ▸; Mueller-Dieckmann *et al.*, 2015 ▸). ID30B was primarily designed to replace and expand the experimental scope of ID14-4 (McCarthy *et al.*, 2009 ▸) and to complement the highly successful tuneable ESRF MX end-stations on ID23-1 (Nurizzo *et al.*, 2006 ▸) and ID29 (de Sanctis *et al.*, 2012 ▸). In particular, a variable focal spot size was considered to be an important objective for two major reasons: for large (∼100–200 µm^3^ sized) homogeneously diffracting crystals of biological macromolecules matching the beam size to the sample size optimizes the signal-to-noise ratios (Evans *et al.*, 2011 ▸); diffraction data collection from small, or large but inhomogeneous, crystals are better suited to a smaller beam size (Bowler *et al.*, 2010 ▸; Evans *et al.*, 2011 ▸; Sanishvili *et al.*, 2008 ▸).

As *in situ* (*i.e.* in crystallization plate or similar) data collection has been shown to be useful (le Maire *et al.*, 2011 ▸; Axford *et al.*, 2012 ▸; Gelin *et al.*, 2015 ▸; Michalska *et al.*, 2015 ▸), particularly for screening small crystals for diffraction quality or collecting data from fragile crystals, it was decided to provide ID30B with an *in situ* plate screening capability based on the highly successful MD2 family of diffractometers (Perrakis *et al.*, 1999 ▸; Cipriani *et al.*, 2007 ▸). A PILATUS3 6M detector with a 1000 µm-thick Si sensor was also purchased to provide suitable X-ray detection at higher energies (>15 keV) thus facilitating atomic-resolution data collection capabilities and, for *in situ* diffraction experiments, providing reduced absorption effects caused by crystallization plates and drops. In addition, and to replace the highly successful but ageing SC3 robots installed on all the ESRF MX beamlines (Cipriani *et al.*, 2006 ▸), a new generation high-density sample changer which combines the Flex robotics (Papp, Felisaz *et al.*, 2017 ▸) with a high-capacity dewar (Nurizzo *et al.*, 2016 ▸) was developed as part of the ID30B project. Since its first commissioning experiments, ID30B has proved to be a versatile MX beamline that expands the experimental capabilities of the JSBG beamline portfolio at the ESRF. Here we describe the beamline’s current configuration and capabilities.

## 2. Beamline overview   

**Figure 1:**
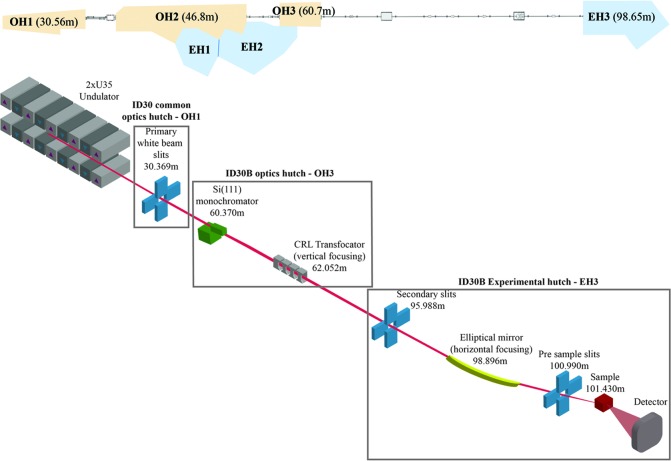
General overview of the ID30 complex (top) and schematic layout of ID30B (bottom). The mean distance of the optical and experimental hutches (top), and the distance of the major components (bottom) in relation to their distance from the source are also shown.

In the canted ID30 straight section, the X-ray source for ID30B consists of two in-air 1.4 m-long undulators with a periodicity of 35 mm (U35) and a minimum operational gap of 11 mm. There are two optical hutches (OH), a common OH1 shared with the ID30A branch, and the ID30B-specific OH3 in which a channel-cut Si(111) monochromator and a transfocator for vertical beam focusing are situated. A long vacuum pipe for ID30B transits the main ID30A optical hutch, OH2, allowing both branches to function independently. A large elliptical mirror required for horizontal focusing at the sample position is situated in the ID30B experimental hutch (EH3). The distance from the source to the sample is over 101 m. A schematic layout of ID30B is shown in Fig. 1 ▸.

### 2.1. OH1 configuration   

The common OH1 houses standard ESRF high-powered primary slits (Marion & Zhang, 2004 ▸), a standard ESRF high-vacuum white beam viewer (WBV), and a photon absorber. The WBV contains a water-cooled optical-grade chemical vapour deposition (CVD) diamond that can be inserted into the X-ray beam using a pneumatic actuator. A Basler acA1300-30gm GigE monochrome camera (Basler AG, Ahrensburg, Germany) is used for visualization *via* a web browser and can also be configured as a counter or X-ray beam position monitor (XBPM) *via* the *LIMA* generic library (Homs *et al.*, 2011 ▸). In addition, the CVD diamond can act as a scattering element for an integrated diode, with adjustable gain settings, which is used to optimally align the primary slits.

### 2.2. OH3 configuration   

OH3 is equipped with a standard ESRF liquid-nitro­gen-cooled Si (111 reflection) channel-cut monochromator for energy selection and a transfocator for the vertical focusing of the X-ray beam. A number of ancillary devices are also installed to facilitate the alignment of the beamline and for X-ray beam diagnostic purposes. These include a WBV, as described above for OH1, installed just before the monochromator about 60 m downstream of the source; a carousel containing two empty slots and a number of calibration foils (Fe, Cu, Pt, Mo and Zr) with a pneumatic activated direct diode read *via* a Keithley picoammeter for monochromator calibration and diagnostic purposes; and a monochromatic beam viewer (MBV) containing a cerium-doped YAG that can be inserted into the X-ray beam using a pneumatic actuator. For the latter device a Basler acA1300-30gm GigE camera is again used for visualization and can again be configured either as a counter or XBPM *via* the *LIMA* generic library (Homs *et al.*, 2011 ▸). This MBV element contains an additional pneumatic actuator containing a foil, the scattering from which is monitored *via* a Keithley picoammeter. This MBV diode is used to optimally align the transfocator.

#### 2.2.1. ID30B channel-cut monochromator   

The ID30B monochromator was previously installed on ID14-4 and has already been described in detail (McCarthy *et al.*, 2009 ▸). The current low-energy limit of the beamline is 6 keV. A number of incremental modifications were carried out on the support of the first Si(111) crystal to try to minimize a slow vertical beam drift, observed as a movement of the X-ray beam position on a scintillator at the sample position during the commissioning of ID30B, and which initially hampered user operation. The beam drift is directly correlated with storage ring current and is symptomatic of thermal changes on the crystal support. The modifications carried out included the unmounting of the crystal and re-machining of the cryo-cooling plates for better thermal contact, the replacement of a ceramic insulation spacer on the weak link ‘pusher’ motor support, and the addition of two heating pads and thermocouples on the crystal support assembly to stabilize the temperature at a constant value (300 K) using a Lakeshore controller. These modifications improved the overall stability and the vertical beam drift was reduced to ∼40–50 µm over 12 h in 200 mA filling modes of the ESRF storage ring. This was corrected for by users routinely (every 1–2 h) running an automatic beam alignment procedure. However, the ESRF now operates in top-up for all storage ring filling modes and we observe much smaller beam drifts (<3 µm over 12 h). These top-up modes will continue to be used in all filling modes post ESRF-Extremely Brilliant Source (EBS) storage ring upgrade (Raimondi, 2016 ▸).

#### 2.2.2. ID30B transfocator   

Vertical focusing of the X-ray beam on ID30B is achieved using parabolic Be compound refractive lenses (CRLs) (Snigirev *et al.*, 1996 ▸). A specially designed transfocator (Vaughan *et al.*, 2011 ▸) contains the required CRLs and is mounted on a motorized four-axis Huber alignment table, similar to that installed for the ID30A MASSIF-1 branch (Bowler *et al.*, 2015 ▸). The transfocator allows for rapidly changing the CRL lens combination (Table 1 ▸) to achieve the vertical focusing size desired at any selected energy. The transfocator has nine mechanical lens holders that can be accurately inserted into the X-ray beam using an alignment guide and pneumatic valves. The first and last holders contain pinholes, which are used to optimally align the transfocator by scanning each motor (translational and rotational) while measuring the intensity observed on the MBV diode (§2.2). Each of the other seven positions contain CRL(s). Currently, the transfocator produces a vertically focused beam size of ∼30 µm (FWHM) across the routinely available energy range of ID30B. Larger vertical beam sizes are achieved by focusing downstream from the sample position by adjusting the number of CRLs in the beam path.

### 2.3. EH3 configuration   

The ID30B experimental hutch, EH3, is situated in the Chartreuse Hall extension of the ESRF and accommodates both the horizontal focusing element and the beamline end-station. EH3 is large as it accommodates a long (1 m) horizontal focusing mirror close to the sample measurement area in order to achieve the required horizontal beam size (20–200 µm). Installation of an ESRF standard X-ray phase-plate setup is foreseen to facilitate the use of polarized resonant scattering in MX anomalous phasing experiments (Schiltz & Bricogne, 2008 ▸). Several other ancillary devices, including beam viewers, slits and X-ray beam conditioning elements (slit box) were also installed to facilitate X-ray beam optimization and alignment procedures.

#### 2.3.1. Horizontal focusing   

ID30B is situated on a high β section of the current ESRF storage ring and provides a horizontal beam size too large to optimally focus using CRLs in OH3 without a significant loss of intensity. Thus to achieve a high-intensity variable beam size in the horizontal direction a large reflecting mirror that can be dynamically bent into an elliptical shape using two actuators attached to either end of the mirror is used (Table 1 ▸). The mirror was fabricated and assembled on its mechanical bending mechanism by WinlightX (Pertuis, France). In order to produce a minimum beam size of 20 µm in the horizontal direction the mirror is centred 2.5 m upstream from the sample position. In order to minimize the transmission of vibrations to the mirror it is mounted in a large high-vacuum vessel mounted on a granite support. The mirror can be translated horizontally and rotated to the required reflecting angle using two motors which slide the top granite table supporting the mirror vessel along low friction plates on a fixed granite support. For optimal focusing, the mirror is set at a reflecting angle of 2.7 mrad. To produce a homogeneous horizontal 20 µm beam size the two actuators can be individually tuned. For larger beam sizes (>20 µm) at the sample position the X-ray beam can be defocused using the same bender mechanism in a synchronous fashion.

The elliptical mirror consists of a silicon substrate with three reflecting stripes (Si, Rh and Pt) on its surface. The mirror height can be adjusted using a single motor coupled to two pushers acting symmetrically on the mirror support *via* feedthrough bellows. This facilitates both movements between reflecting stripes and those required, due to the vertical displacement of the X-ray beam by the channel-cut monochromator, upon changing energy. The Rh stripe is used by default as it provides >93% reflectivity for the current operating range (6 to 20 keV). However, the Si stripe is particularly useful at lower energies (<8 keV), removing contaminating higher harmonic X-rays while maintaining a high intensity (>1 × 10^12^ photons s^−1^ mm^−2^ at 6 keV). As Rh reflectivity significantly reduces at energies >20 keV the Pt stripe facilitates data collection at even higher energies should this be required at a later stage of the beamline’s development.

Two MBV assemblies similar to that described above in OH3 (§2.2) are installed in EH3. The first contains only the beam viewer option and is primarily used for diagnostic purposes. The second assembly contains a retractable scattering foil diode, and is installed after the phase-plate vessel and a pair of beam-defining secondary slits (ss2) (JJ-XRAY, Lyngby, Denmark) installed to facilitate the optimal alignment of the elliptical mirror, by enabling a narrow horizontal slit beam to be scanned across the mirror while recording the coordinates of the X-ray beam at the sample position. This setup has proved to be very reliable and we are currently implementing software routines for optimal mirror alignment.

Downstream of the mirror is a high-vacuum attenuator box containing a total of eight foils: two pyrocarbon (1 and 2 mm in thickness) and six Al (0.1, 0.2, 0.35, 0.5, 1 and 1.5 mm thickness, respectively). For insertion into the X-ray beam, each foil is mounted on an individual pneumatic actuator and the composition of the foils in the attenuator box chosen to best match the attenuation required across the energy range of ID30B.

### 2.4. EH3 sample environment   

**Figure 2:**
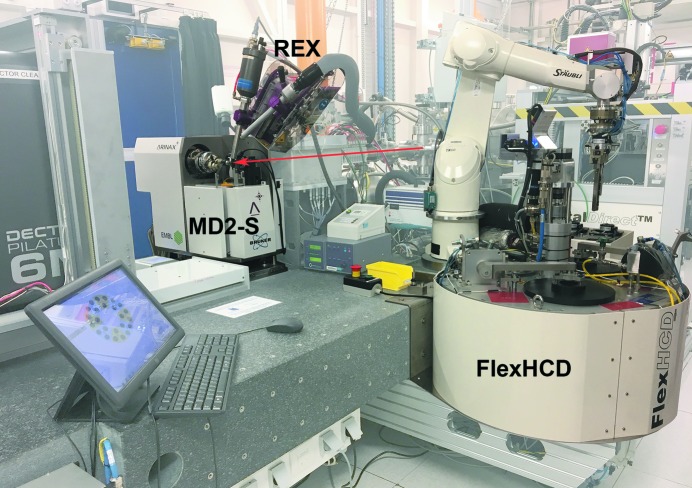
Photograph of the ID30B experimental hutch highlighting the MD2S diffractometer, REX rapid nozzle exchanger and FlexHCD sample changer. The beam path is highlighted as a red arrow.

The ID30B sample environment is composed of a MD2S diffractometer (Arinax, Moirans, France), a PILATUS3 6M detector with a 1 mm-thick Si sensor [Dectris, Baden-Dättwil, Switzerland (Broennimann *et al.*, 2006 ▸)] mounted on a fast translation table, and a FlexHCD robotic sample changer (Fig. 2 ▸). In addition, a 700 series cryo-stream (Oxford Cryostreams, Oxford, UK) with annealing device (Giraud *et al.*, 2009 ▸) is mounted on a REX rapid nozzle exchanger (Arinax, Moirans, France). Similar to the other MX beamlines at the ESRF the whole experimental setup is fixed on a large granite table to facilitate the simultaneous alignment of all elements to the X-ray beam position. The table is mounted on three motorized vertical supports and can be translated in the horizontal direction using two additional motors. These motors are in a closed loop and can be moved independently, or as a series of pseudo motors for controlling the table height, translation, roll, pitch and yaw. This setup facilitates an easy alignment of the experimental setup with the X-ray beam, especially after energy changes as channel-cut monochromators displace the X-ray beam vertically during this procedure.

**Figure 3:**
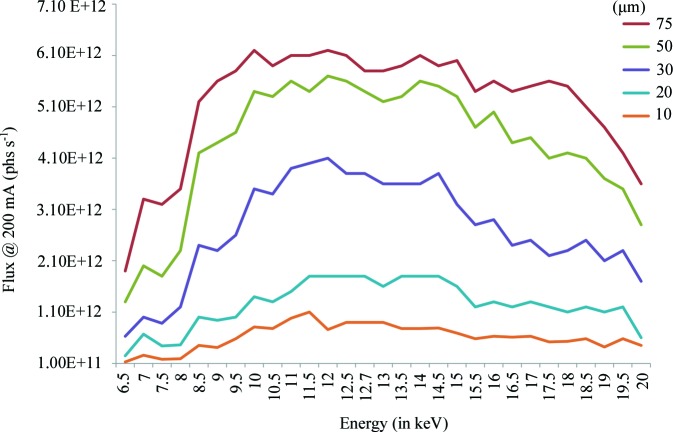
Photon flux (photons s^−1^) at the sample position as a function of photon energy and beam-defining aperture used at 200 mA storage ring curent.

The first element on the experimental table is a high-vacuum slit box containing a single set of beam-defining/cleaning slits (JJ-XRAY, Lyngby, Denmark) followed by two beam diagnostic elements separated by a fast (∼70 ms per 90°) rotary shutter containing a large opening. The fast shutter is connected to the MD2S diffractometer to allow synchronization with its goniometer axis. Both beam diagnostic elements are comprised of a cerium-doped YAG screen with a Basler acA1300-30gm GigE camera for visualization of the X-ray beam and a 125 µm-thick Kapton scattering foil diode mounted on a rotary table for easy insertion or retraction in the X-ray beam. During user operation, the diodes remain in the X-ray beam and are read using a Keithley picoammeter. The readings from these diodes are regularly calibrated at different energies and aperture settings against a standardized control diode placed at the sample position. These readings provide continuous real-time flux measurements during experiments and are relatively constant across the energy range (6–20 keV) available on ID30B (Fig. 3 ▸).

#### 2.4.1. The MD2S diffractometer   

**Figure 4:**
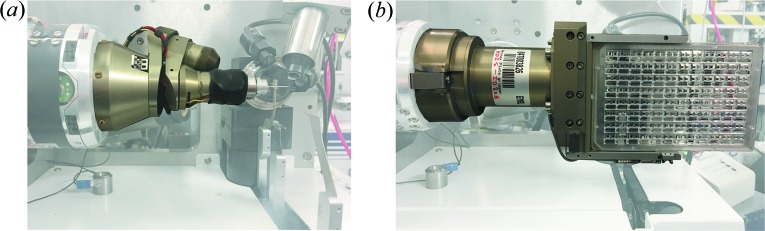
The MD2S on ID30B in (*a*) Mini-kappa (MK3) and (*b*) *in situ* plate holder goniometer head configuration.

The MD2S diffractometer, the first device of this type installed on an MX beamline, has a maximum goniometer axis rotation speed of 720° s^−1^, and a Mini-Kappa goniometer head (MK3) (Cipriani *et al.*, 2007 ▸; Brockhauser *et al.*, 2011 ▸, 2013 ▸) is mounted to provide crystal realignment with a sphere of confusion of <1.5 µm (Fig. 4 ▸ *a*). An AXAS-A X-ray fluorescence detector (KETEK, Munich, Germany) on a retractable pneumatic table has also been fully integrated into the MD2S. An additional digital I/O card was installed in the MD2S control electronics rack for direct detector control thus allowing a gated triggering of the PILATUS3 6M detector. This has enabled the development of fast continuous mesh scanning data collection protocols (see movie S1 of the supporting information). The MD2S control software runs on a Windows PC and is based on the *JLib* library (EMBLEM Technology Transfer GmbH, Heidelberg, Germany; http://software.embl-em.de). The MD2S can be controlled using a graphical user interface (GUI) or remotely by a socket server using the Exporter protocol of *JLib*. The Prosilica GC655C GigE on-axis video camera of the MD2S is read using the *LIMA* generic library for high-throughput image acquisition (Homs *et al.*, 2011 ▸).

The MD2S diffractometer is currently equipped with a penta-aperture (75, 50, 30, 20 and 10 µm in diameter) that can be easily inserted to adjust the beam size as experimentally required. A second penta-aperture set primarily for larger beam sizes (150, 100, 75, 50 and 20 µm in diameter) is available on request. The MD2S is an evolution of the standard MD2 diffractometers installed on other ESRF MX beamlines such as ID29 (de Sanctis *et al.*, 2012 ▸). It combines a large-range goniometer axis translation stage with a new beam-conditioning device originally developed for the MD3 on the EMBL P14 beamline at PETRA-III. This allows the diffractometer’s beam-cleaning capillary and beamstop to move independently. The current beamstop is 400 µm in diameter and its distance from the crystal can be varied from 9 to 60 mm with the larger distances facilitating the collection of very low resolution reflections. A larger beamstop, 800 µm in diameter, is available when required.

#### 2.4.2. *In situ* data collection   

**Figure 5:**
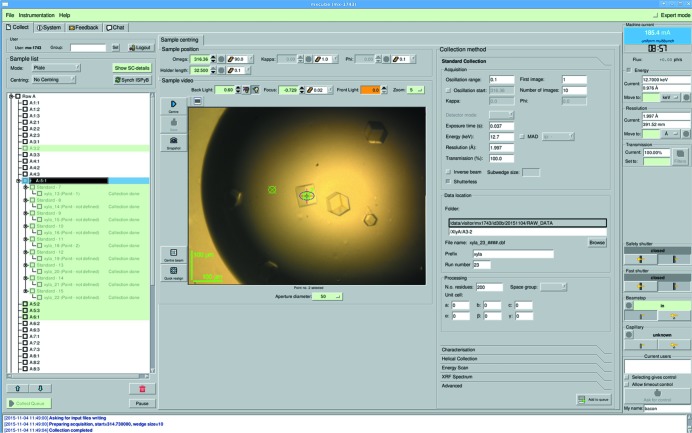
*In situ* data collection on ID30B as implemented in *MxCuBE*.

The MD2S was specially designed to allow *in situ* data collection from crystallization plates. For this, the cryo-stream is first moved away from the sample environment using the REX rapid nozzle changer (Fig. 2 ▸). The cleaning capillary is also removed and disabled, while the beam-defining aperture is replaced with a 20 µm canon aperture (a combined aperture and beam-cleaning capillary). The MK3 goniometer head (Cipriani *et al.*, 2007 ▸, Brockhauser *et al.*, 2011 ▸, 2013 ▸) can then be unmounted and replaced by a new type of crystal plate holder incorporating a novel ‘quick lock’ mechanism to facilitate the swap between MK3 and plate holder goniometer heads that was specially developed for ID30B (Fig. 4 ▸ *b*). The plate holder can accommodate low-profile SBS footprint plates (127.76 × 85.48 × 8.0 mm) and is currently configured to accommodate CrystalDirect™ (Cipriani *et al.*, 2012 ▸; Zander *et al.*, 2016 ▸), CrystalQuick™X and *In Situ*-1™ crystallization plates. Due to space constraints induced when measuring samples in crystallization plates a number of interlocks have also been configured to prevent potential collisions. Using this set-up, oscillation data (±20^o^) can successfully be collected from *in situ* crystals and this functionality has now been implemented in *MxCuBE* (Fig. 5 ▸). It takes ∼40 min to exchange goniometer heads and configure the MD2S for *in situ* data collection. The plates are manually mounted on the beamline and we currently schedule one shift (8 h) every other week for *in situ* experiments, but remote experiments can be carried out upon request.

### 2.5. X-ray fluorescence measurements   

A Ketek AXAS-A X-ray fluorescence detector (Ketek, Munich, Germany) connected to a Bruker MultiMax signal processing unit (Bruker AXS, Karlsruhe, Germany) is used for X-ray absorption near-edge structure (XANES) measurements. The X-ray fluorescence region of interest (ROI) for a particular element’s absorption edge is calculated and programmed *via* an RS-232 serial line connection. As for all energy-tuneable MX beamlines (ID23-1, ID29 and ID30B) at the ESRF, the ROI signal, using the MultiMax TTL output, is registered synchronously with the energy, during a continuous motion of the monochromator by the multipurpose unit for synchronization, sequencing and triggering (MUSST) module developed at the ESRF. Thus, a complete XANES scan, 100 eV around the theoretical absorption edge, is typically recorded in 20–30 s. As previously described (Leonard *et al.*, 2009 ▸), this setup also allows a complete X-ray fluorescence (XRF) spectrum to be recorded *via* the serial line connection and to be subsequently analysed using *PyMCA* (Solé *et al.*, 2007 ▸).

### 2.6. The FlexHCD sample changer   

ID30B started user operation in June 2015 with a SC3 sample changer (Cipriani *et al.*, 2006 ▸) installed. However, a new generation sample changer was clearly required to overcome the limited sample capacity of the SC3, to allow the use of sample holders formats used elsewhere (*i.e.* Uni-Puck; http://smb.slac.stanford.edu/robosync/Universal_Puck/) and to accommodate new sample holding formats being developed, such as miniSPINE (Papp, Rossi *et al.*, 2017 ▸). A decision was therefore taken to combine the Flex robotic technology developed by the EMBL-Grenoble instrumentation team (Papp, Felisaz *et al.*, 2017 ▸) with the ESRF HCD developed as part of the MASSIF-1 (ID30A-1) project (Nurizzo *et al.*, 2016 ▸). This new sample changer, the FlexHCD, combines the versatility and reliability of a Stäubli TX60L six-axis industrial robot (Stäubli Faverges SCA, Faverges, France) with a robust, large-capacity storage dewar, a fundamental requirement for modern MX beamlines.

**Figure 6:**
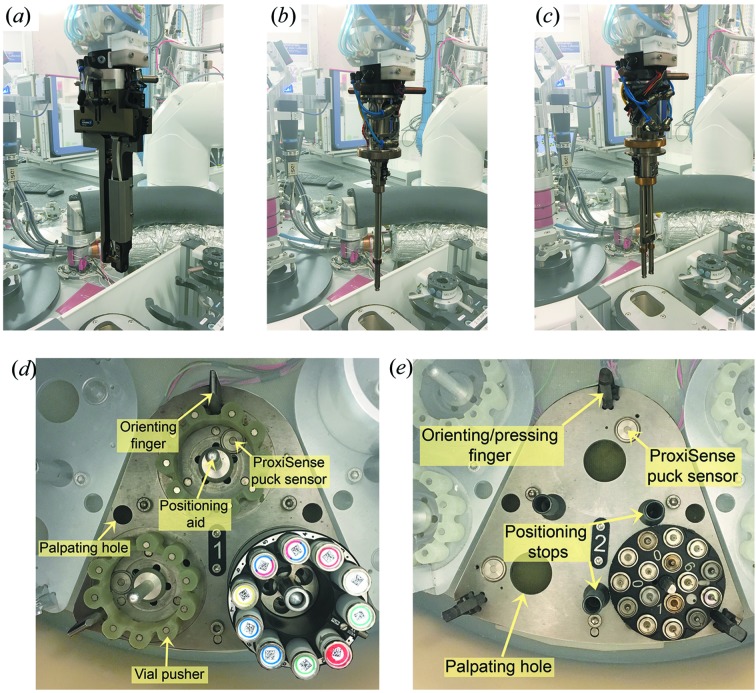
Three types of FlexHCD grippers are currently available on ID30B: (*a*) a modified IRELEC flipping gripper; EMBL-designed single (*b*) and double (*c*) SPINEplus grippers. The HCD was modified to allow storage of samples in (*d*) SC3 or (*e*) Uni-Puck storage formats. Orientation and positioning aids help facilitate puck loading by users. ProxiSense puck detection sensors are used for puck type recognition, and to ensure the pucks are correctly positioned and are not inadvertently removed during sample loading.

The robotic arm of the FlexHCD is equipped with a tool changer and tool storage rack that can accommodate five gripper types. The robot on ID30B is currently equipped with a calibration tool for automatic robot alignments (Papp, Felisaz* et al.*, 2017 ▸), an IRELEC flipping gripper (Jacquamet *et al.*, 2009 ▸) for samples stored in SC3 format pucks (Cipriani *et al.*, 2006 ▸), and EMBL-designed single and double SPINEplus grippers (Papp, Felisaz *et al.*, 2017 ▸) for samples stored in Uni-Puck format (Figs. 6 ▸ *a*, 6*b* and 6*c*). Only SPINE standard sample holders (Cipriani *et al.*, 2006 ▸) are compatible with the SPINEplus grippers.

In order to integrate both SC3 and Uni-Puck format pucks on ID30B, the HCD was significantly modified compared with the version installed on MASSIF-1 (Nurizzo *et al.*, 2016 ▸). First, two types of segment plates were designed. For SC3 format pucks a ring-shaped plastic plug with magnetic disks pushes the vials up about 15 mm to ensure that the IRELEC gripper has access to the vials (Fig. 6 ▸ *d*). The magnetic disks guarantee that the vials are held in position and are refilled with liquid nitro­gen after sample loading. To facilitate loading of the pucks an orienting finger that fits in the SC3 puck groove, coupled with a positioning aid, help to align the pucks correctly in the HCD. The second type of segment plate has a Uni-Puck footprint (Fig. 6 ▸ *e*). Again, an orienting/pressing finger and positioning stops facilitate the precise loading of Uni-Pucks by users. Each puck position (SC3 and Uni-Puck) is equipped with a ProxiSense puck sensor and readout electronics (Papp, Felisaz *et al.*, 2017 ▸), allowing detection of the presence and correct positioning of pucks in the HCD. Secondly, the positional accuracy of the HCD rotation axis was improved and migrated to a Modbus electronic module (WAGO Contact SAS, Roissy, France) control system. These modifications were carried out to ensure the reliable robotic handling of samples in Uni-Puck format and to facilitate the potential use of high-density sample holders in the future (Papp, Rossi *et al.*, 2017 ▸). On ID30B the HCD is configured to accommodate 12 SC3 pucks and 11 Uni-pucks, but this layout can be adapted by changing segment plates. The dewar filling and exhaust ports of the HCD have been modified such that the filling port is now used for both liquid-nitro­gen filling and venting. The original venting port was equipped with a pneumatic port and is now used to cool the SPINEplus grippers between operations. The liquid nitro­gen in the HCD is maintained at a constant level using detection monitors coupled to valves for refilling by an external reserve tank.

The HCD contains two large ports, one for manual user loading of sample containers (SC3 or Uni-Puck), the second for robot access. A GigE connected UI-5240CP-M-GL camera (IDS Imaging Development Systems GmbH, Obersulm, Germany) and two MS-4Xi (Omron Microscan Systems Inc, Renton, WA, USA) have been integrated for robotic diagnostics and sample bar code reading, respectively. During sample loading, or unloading, a series of images are taken and processed to determine whether a sample has been properly handled. The image processing algorithm was further developed to detect and correct for small variations in the sample positioning in the grippers. If a sample is detected as too far from predefined specifications, the sample transfer can be refused and returned to the dewar or placed in a special receptacle in the HCD for later recovery.

The high-level software control of the FlexHCD system on ID30B runs on a Windows PC and is based on the *JLib* library software suite (EMBLEM Technology Transfer GmbH, Heidelberg, Germany; http://software.embl-em.de), encompassing a *StaubCom* server for robotic movements, a WAGO controller for HCD carousel rotations, a pr­oxy sense card for sample holder detection, and all image acquisition and processing software. The FlexHCD software can be controlled using a dedicated GUI or remotely by a socket server using the Exporter protocol of *JLib*.

### 2.7. Beamline control software   

The individual motors required to align the optical elements, move the experimental table and detector translation table as well as open and close the millisecond fast shutter are all controlled at the lowest level by ESRF-developed IcePAP electronics (Janvier *et al.*, 2013 ▸). All pneumatic devices on ID30B are driven *via* WAGO-I/O-System 750 control modules (WAGO Contact SAS, Roissy, France), which are also used to monitor thermocouples. The scattering foil diodes on ID30B are read *via* Keithley picoammeters.

All XBPM Basler acA1300-30gm GigE cameras and the MD2S sample camera on ID30B can be accessed *via* the *LIMA* generic library (Homs *et al.*, 2011 ▸) and viewed in a web browser. All motors, apart from those of the MD2S diffractometer and FlexHCD sample changer, are controlled on a higher level using *BeamLine Instrumentation Support Software* (*BLISS*), the new ESRF system for experiment control, which is a Python-based open source project [https://gitlab.esrf.fr/bliss/bliss (Guijarro *et al.*, 2017 ▸)]. A number of pseudo motors, such as slit gaps and offsets, table height, translation, roll, pitch and tilt are configured in *BLISS*, which also provides procedures for the automatic alignment of the experimental environment to the X-ray beam. All the beamline thermocouples and diodes are configured to be read using *BLISS*, which additionally provides, *via* a unified web-based application shell, a graphical scanning functionality to facilitate beamline alignment, develop automated alignment routines, and diagnose beamline-specific problems.

As for all the ESRF MX beamlines, *MxCuBE*, the *Macromolecular Crystallography Customized Beamline Environment* software [http://mxcube.github.io/mxcube (Gabadinho *et al.*, 2010 ▸)], provides the user interface to control instruments and procedures required for a diffraction experiment. These include calls to the underlying *BLISS* control system for the execution of procedures such as energy changes, alignments and various scans; loading and unloading of samples by the FlexHCD; and MD2S data collection protocols using the Exporter protocol of *JLib*. The PILATUS3 6M detector control software runs on a dedicated Linux computer and is triggered for data collection *via* the *LIMA* generic library PILATUS implementation, sending commands to the Dectris *camserver*. For automated data acquisition protocols, as already described for MASSIF-1 (Svensson *et al.*, 2015 ▸), we use the *Beamline Expert System* (*BES*), a customized version of *Passerelle EDM* (https://supportsquare.io/portfolio/products/) running on a central computing cluster. Meanwhile, all experimental planning and final data collection parameters are stored in the ISPyB database for MX with results of automatic data processing (Monaco *et al.*, 2013 ▸) and analyses of characterization and *BES* workflows displayed in *ExiMX* (http://exi.esrf.fr/), the ESRF user interface to ISPyB (Delagenière *et al.*, 2011 ▸).

## 3. Results   

### 3.1. Data collection at cryogenic temperature   

On ID30B, diffraction data are routinely collected at 100 K from samples mounted in SPINE standard pins [see http://www.spineurope.org for details (Cipriani *et al.*, 2006 ▸)]. For example, *Thaumatococcus daniellii* thaumatin was crystallized and cryoprotected as previously described (Nanao *et al.*, 2005 ▸). Diffraction data were subsequently collected from a single crystal using a radiation damage optimized strategy calculated by *BEST* (Bourenkov & Popov, 2010 ▸) as implemented in *EDNA* (Incardona *et al.*, 2009 ▸). This data set was processed with *XDS* (Kabsch, 2010 ▸) and *AIMLESS* (Evans *et al.*, 2011 ▸) using an automatic data processing pipeline (Monaco *et al.*, 2013 ▸) and the results downloaded from *ExiMX*. The structure was further refined using *REFMAC* (Murshudov *et al.*, 2011 ▸), waters added using *ARPwarp* (Lamzin *et al.*, 2012 ▸) and the results visually inspected with *Coot* (Emsley *et al.*, 2010 ▸), all as implemented in *CCP4* (Winn *et al.*, 2011 ▸). A summary of the crystallographic information is shown in Table 2 ▸.

To further illustrate the capabilities of the beamline, several single-wavelength anomalous diffraction (SAD) experimental phasing measurements were carried out on three test proteins. Firstly, *Bacillus thermoproteolyticus* thermolysin was crystallized and cryoprotected as previously described (Zander *et al.*, 2015 ▸). Diffraction data from a single flash-frozen crystal were collected at 100 K using an X-ray beam size of 30 µm^2^ with a flux of 2.3 × 10^11^ photons s^−1^ at the peak of the Zn *K* absorption edge (λ = 1.282 Å). Next, a seleno­methione-derivative of the feruloyl esterase module of xylanase 10B from *Clostridium thermocellum* (FAE) was crystallized and cryoprotected as previously described (Prates *et al.*, 2001 ▸). Diffraction data from a single flash-frozen crystal were collected at 100 K using an X-ray beam size of 30 µm^2^ with a flux of 3.3 × 10^11^ photons s^−1^ at the peak of the Se *K* absorption edge (λ = 0.9792 Å). Lastly, porcine trypsin was crystallized and cryoprotected as previously described (Nanao *et al.*, 2005 ▸). Diffraction data from a single flash-frozen crystal were collected at 100 K using an X-ray beam size of 30 µm^2^ with a flux of ∼7 × 10^10^ photons s^−1^. Here, a longer wavelength of 6 keV (λ = 2.066 Å) and the Si-stripe of the horizontal mirror were used to optimize the anomalous signal from the intrinsic sulfur atoms present in the crystal and two consecutive low-dose data sets of 360^o^ total rotation were collected; for the first data set the crystal was aligned along c* using the MK3 goniometer head to ensure that Bijvoet pairs were collected simultaneously, while for the second the crystal was in a random orientation. This strategy is particularly recommended for S-SAD phasing experiments (Brockhauser *et al.*, 2013 ▸).

All data were processed and scaled using the *XDS* suite (Kabsch, 2010 ▸) and *AIMLESS* (Evans *et al.*, 2011 ▸), and are of very high quality (Table 3 ▸). Structure solution was carried out using the SAD phasing technique as implemented in the *CRANK2* pipeline (Skubák & Pannu, 2013 ▸; Skubák *et al.*, 2018 ▸), resulting in clearly interpretable electron density maps: for thermolysin, 306 residues out of a total of 315 were automatically built (*R*/*R*_free_ = 28.6/30.3%); for FAE, 284 residues out of a total of 297 were automatically built (*R*/*R*_free_ = 25.5/28.1%); for trypsin, 224 residues out of a total of 246 were automatically built (*R*/*R*_free_ = 30.7/35.6%). All three structures were then refined using the PDB_redo server (Joosten *et al.*, 2014 ▸) and model building carried out in *Coot* (Emsley *et al.*, 2010 ▸). A summary of all crystallographic information is shown in Table 3 ▸.

More complex experiments such as ‘MeshAndCollect’ (Zander *et al.*, 2015 ▸) have benefitted from the development of the rapid mesh scanning protocol (see movie S1 of the supporting information) on ID30B. As an example this method was recently used to determine the structure of an important human hormone receptor, Adinonectin receptor 2 (Vasiliauskaité-Brooks *et al.*, 2017 ▸). The fast mesh scanning option combined with the reliability of the FlexHCD has allowed the implementation of fully automatic MXPress workflows (Svensson *et al.*, 2015 ▸) on ID30B. For such workflows, processing time per sample currently varies from less than 4 to around 8 min depending on the complexity of the workflow executed.

### 3.2. *In situ* diffraction experiments   

The ID30B *in situ* data collection setup (§2.4.2) is compatible both with standard and raster-based data collections between 6 and 20 keV. While the total rotation range is limited due to the restricted sample environment, complete data sets can be collected from large single crystals with high-symmetry space groups lying in favourable orientations. In the case of smaller, lower-symmetry crystals the most successful approach is to create a complete data set from multiple partial data sets collected from different crystals. To validate our *in situ* setup a number of data sets of thaumatin crystals grown in CrystalDirect™ (Cipriani *et al.*, 2012 ▸; Zander *et al.*, 2016 ▸) (using a flux of ∼8 × 10^10^ photons s^−1^ with a 20 µm^2^ cannon aperture; 20 ms exposure per 0.1^o^ oscillation; total oscillation range of 50°) were collected at 12.7 and 17.5 keV with an ESRF storage ring current of 4 × 10 mA. The most complete data set from the automatic data processing pipeline (Monaco *et al.*, 2013 ▸) at each energy was downloaded from *ExiMX* and the crystal structures refined to 1.5 Å resolution. Crystallographic information is shown in Table 2 ▸.

## 4. Conclusions and discussion   

ESRF beamline ID30B has been in full user operation, including remote access, since June 2015 and, at the time of writing, data collected at the beamline has resulted in 83 PDB depositions and 62 publications. A significant amount of time during the first two years was dedicated to the optimization of the experimental setup including commissioning of the FlexHCD sample changer and the *in situ* plate holder. The FlexHCD sample loading/unloading time is currently <20 s, and <12/14 s using the IRELEC flipping gripper and SPINEplus single/double gripper, respectively. This equates to a sample-to-sample exchange time, when all preparative (changing the MD2S to a sample transfer configuration, and moving the detector to a safe distance as well as protecting its sensor area with a cover) and diagnostic (multiple images of the grippers are taken and processed during loading/unloading cycles) steps are included, of <60 s, and <50/40 s using the IRELEC flipping gripper and SPINEplus single/double gripper, respectively. In 2017 the FlexHCD processed 13166 and 3253 samples from SC3 and Uni-puck storage containers, respectively, with overall rates of sample loss and user intervention of <0.01% and <1%. We are currently exploring ways to further improve the speed, reliability and automatic error recovery of the FlexHCD.

Users now routinely execute automated MXPress data collection protocols and we expect beamline productivity to increase as users take more advantage of these. Moreover, we will soon complement the currently available workflows with automated experimental phasing data collection protocols, some based on the use of the MK3. Other instrument developments will include the evaluation of a CrystalDirect™ harvester (Cipriani *et al.*, 2012 ▸; Zander *et al.*, 2016 ▸) recently installed on the beamline for feasibility studies, the development of a plate-changing tool for the Flex robot, and use of the REX nozzle changer to facilitate dehydration experiments (Sanchez-Weatherby *et al.*, 2009 ▸; Russi *et al.*, 2011 ▸) and room-temperature data collection using a HC-Lab humidity Controller (Arinax, Moirans, France).

## Supplementary Material

Diffraction-based X-ray centring using a continuous two-dimensional mesh scan as implemented in MxCuBE on ID30B at the ESRF.. DOI: 10.1107/S1600577518007166/ig5055sup1.mp4

## Figures and Tables

**Table 1:** Summary of beamline ID30B parameters

Beamline name	ID30B
Source type	2 × 1.4 m in-air U35 undulators (minimum gap 11 mm)
Monochromator	Si(111)
Energy range (keV)	6.0–20.0
Vertical focusing elements	One-dimensional refractive lenses in transfocator
Lens material	Be
Number × radii of parabola apex (µm)	1 × 500, 2 × 500, 4 × 500, 8 × 500, 1 × 1000, 2 × 1000, 1 × 1500
Horizontal focusing element	Bendable tangential elliptical cylinder mirror
Dimensions (mm)	1100 × 85 × 40
Glancing angle (mrad)	2.7
Surface coatings	Si, Rh and Pt
Surface roughness (Å)	<3
Slope error (µrad)	<0.5
Average tangential radius at focus (m)	3200
Focused beam size (FWHM, H × V) (µm)	20 × 30
Focused beam flux (photons s^−1^) at 12.7 keV	5.5 × 10^12^ at 200 mA
Diffractometer	MD2S
Sample mounting	FlexHCD
Detector type	CMOS Hybrid Pixel
Detector model	Dectris PILATUS 6M (1000 µm Si sensor)

**Table 2:** Data collection and refinement statistics of thaumatin collected at 100 and 293 K (*in situ*) Statistics for the highest-resolution shell are shown in parentheses.

Data collection			
Energy (keV)	12.67	12.7	17.5
Temperature (K)	100	293 (*in situ*)	293 (*in situ*)
Resolution range (Å)	50–1.08 (1.12–1.08)	50–1.5 (1.53–1.5)	50–1.5 (1.53–1.5)
Space group	*P*4_1_2_1_2	*P*4_1_2_1_2	*P*4_1_2_1_2
Unit cell (Å, °)	58.0, 58.0, 150.7, 90, 90, 90	58.6, 58.6, 151.6, 90, 90, 90	58.6, 58.6, 151.5 90, 90, 90
Unique reflections	99222 (6841)	42124 (2031)	39794 (1988)
Multiplicity	3.6 (1.6)	3.6 (3.5)	3.4 (3.4)
Completeness (%)	89.8 (63.5)	98.2 (98.4)	92.6 (96.7)
Mean 〈*I*/σ(*I*)〉	11.8 (0.8)	5.7 (1.0)	7.2 (1.3)
Wilson *B* factor (Å^2^)	11.4	15.2	12.4
(*I*/σ)^asymptotic^ †	13.3	8.2	12.3
*R*_p.i.m._ (%)‡	2.9 (54.4)	7.1 (71.2)	5.7 (51.8)
CC*	0.996 (0.857)	0.99 (0.39)	0.99 (0.6)

Structure refinement			
*R*_work_ (%)	14.8 (47.0)	13.0 (29.6)	13.8 (26.4)
*R*_free_ (%)	16.6 (45.4)	16.7 (35.3)	16.7 (29.0)
No. of non-H atoms	1806	1724	1725
Macromolecule	1600	1594	1594
Tartrate/glycerol	34	10	10
Water	172	120	121
R.m.s.d. (bonds, Å)	0.017	0.014	0.01
R.m.s.d. (angles, ^o^)	1.8	1.6	1.4
Ramachandran plot (%)			
Favored	98.6	98.6	98.6
Allowed	1.4	1.4	1.4
Average *B* factor (Å^2^)			
Macromolecules	16.8	21.7	19.5
Tartrate/glycerol	29.0	19.7	17.5
Solvent	33.0	22.4	30.7
Diffraction data	https://doi.org/10.15785/SBGRID/544	https://doi.org/10.15785/SBGRID/546	https://doi.org/10.15785/SBGRID/547
PDB code	6fj6	6fj8	6fj9

† (*I*/σ)^asymptotic^: as reported in *XDS* (Diederichs, 2010 ▸).

‡ *R*_pim_: precision-weighted merging *R*-factor (Weiss, 2001 ▸).

**Table 3:** Data collection and phasing of trypsin by S-SAD, thermolysin by Zn-SAD and FAE by Se-SAD Statistics for the highest-resolution shell are shown in parentheses.

Protein	Trypsin	Thermolysin	FAE
Data collection			
Energy (keV)	6	9.672	12.662
Resolution range (Å)	50.0–2.2 (2.27–2.2)	50–1.43 (1.45–1.43)	50–1.7 (1.73–1.71)
Space group	*P*2_1_2_1_2_1_	*P*6_1_22	*P*4_1_2_1_2
Unit cell (Å, °)	60.0, 64.1, 69.7 90, 90, 90	92.7, 92.7, 128.6 90, 90, 120	112.0, 112.0, 65.9 90, 90, 90
Unique reflections	13845 (1057)	60230 (2 379)	207445 (11053)
Anom. multiplicity	11.8 (6.5)	2.8 (2.0)	2.4 (2.3)
Anom. completeness (%)	97.2 (85.8)	96.7 (60.6)	96.3 (96.1)
Mean 〈*I*/σ(*I*)〉	24.9 (10.4)	13.6 (3.7)	11.2 (0.9)
Wilson *B* factor (Å^2^)	15.1	11.3	24.2
(*I*/σ)^asymptotic^ †	12.1	9.8	19.0
*R*_p.i.m._ (%)‡	2.4 (7.4)	4.2 (13.7)	4.1 (84.1)
CC*	0.997 (0.98)	0.99 (0.93)	0.997 (0.318)
Anom. mid-slope§	1.136	1.224	1.293
SigAno	1.23 (0.76)	1.48 (1.57)	1.44 (0.7)

Structure refinement			
*R*_work_ (%)	17.6 (19.7)	14.5 (19.0)	18.3 (36.4)
*R*_free_ (%)	22.0 (27.5)	16.4 (19.6)	20.1 (37.1)
No. of non-H atoms	1830	2948	2358
Macromolecules	1636	2561	2232
Water	170	362	50
Ligand/ion/glycerol	24		
R.m.s.d. (bonds, Å)	0.008	0.017	0.02
R.m.s.d. (angles, ^o^)	1.3	1.7	1.7
Ramachandran plot (%)			
Favored	97.3	97.3	97.9
Allowed	2.7	2.7	2.1
Average *B* factor (Å^2^)			
Macromolecules	10.2	8.7	20.7
Ligand/ion/glycerol	36.0	32.6	
Solvent	26.6	27.6	23.5
Diffraction images	https://doi.org/10.15785/SBGRID/541	https://doi.org/10.15785/SBGRID/542	https://doi.org/10.15785/SBGRID/543
PDB code	6fid	6fj2	6fj4

† (*I*/σ)^asymptotic^: as reported in *XDS* (Diederichs, 2010 ▸).

‡ *R*_pim_: precision-weighted merging *R*-factor (Weiss, 2001 ▸).

§ Anom. mid-slope: mid-slope of anomalous normal probability in *AIMLESS* (Evans *et al.*, 2011 ▸).
